# Regional neural functional efficiency across schizophrenia, bipolar disorder, and major depressive disorder: a transdiagnostic resting-state fMRI study

**DOI:** 10.1017/S0033291724001685

**Published:** 2024-11

**Authors:** Jun Yang, Zhening Liu, Yunzhi Pan, Zebin Fan, Yixin Cheng, Feiwen Wang, Fuping Sun, Guowei Wu, Xuan Ouyang, Haojuan Tao, Jie Yang, Lena Palaniyappan

**Affiliations:** 1 Department of Psychiatry, National Clinical Research Center for Mental Disorders, and National Center for Mental Disorders, The Second Xiangya Hospital of Central South University, Changsha, Hunan, China; 2 Department of Psychiatry, Douglas Mental Health University Institute, McGill University, Montreal, Quebec, Canada; 3 Department of Medical Biophysics, Schulich School of Medicine and Dentistry, Western University, London, Ontario, Canada; 4 Schulich School of Medicine and Dentistry, Robarts Research Institute, Western University, London, Ontario, Canada

**Keywords:** activity, calcium, connectivity, dynamic, glutamate, potassium, static, variability

## Abstract

**Background:**

Major psychiatric disorders (MPDs) are delineated by distinct clinical features. However, overlapping symptoms and transdiagnostic effectiveness of medications have challenged the traditional diagnostic categorisation. We investigate if there are shared and illness-specific disruptions in the regional functional efficiency (RFE) of the brain across these disorders.

**Methods:**

We included 364 participants (118 schizophrenia [SCZ], 80 bipolar disorder [BD], 91 major depressive disorder [MDD], and 75 healthy controls [HCs]). Resting-state fMRI was used to caclulate the RFE based on the static amplitude of low-frequency fluctuation, regional homogeneity, and degree centrality and corresponding dynamic measures indicating variability over time. We used principal component analysis to obtain static and dynamic RFE values. We conducted functional and genetic annotation and enrichment analysis based on abnormal RFE profiles.

**Results:**

SCZ showed higher static RFE in the cortico-striatal regions and excessive variability in the cortico-limbic regions. SCZ and MDD shared lower static RFE with higher dynamic RFE in sensorimotor regions than BD and HCs. We observed association between static RFE abnormalities with reward and sensorimotor functions and dynamic RFE abnormalities with sensorimotor functions. Differential spatial expression of genes related to glutamatergic synapse and calcium/cAMP signaling was more likely in the regions with aberrant RFE.

**Conclusions:**

SCZ shares more regions with disrupted functional integrity, especially in sensorimotor regions, with MDD rather than BD. The neural patterns of these transdiagnostic changes appear to be potentially driven by gene expression variations relating to glutamatergic synapses and calcium/cAMP signaling. The aberrant sensorimotor, cortico-striatal, and cortico-limbic integrity may collectively underlie neurobiological mechanisms of MPDs.

## Introduction

Long defined as distinct diagnostic categories, three major psychiatric disorders (MPDs) of adult life – schizophrenia (SCZ), bipolar disorder (BD), and major depressive disorder (MDD), share many common features. Psychotic symptoms (delusions, hallucinations, disorganized thinking, and psychomotor behavior) and affective features are present in all three disorders; episodic nature (relapses and remissions) and long-term recurrences typify the course for many patients with these diagnoses (Lieberman & First, 2018) while pharmacological approaches that alleviate these symptoms cut across the traditional diagnostic categories (Aminoff et al., 2022; Dai et al., 2018; Heslin & Young, 2018). Recent neurobiological research also indicates essential overlaps of genetic, cellular and molecular abnormalities among these three disorders (Brosch et al., 2022; Grotzinger et al., 2022; Pinto, Moulin, & Amaral, 2017). Thus, converging evidence from clinical observations to neurobiology suggest common core features among major psychiatric disorders. This naturally leads us to ask if there is a pattern of shared dysfunction in the neural physiology of MPDs.

Several neuroimaging studies have indicated a notable overlap among the MPDs in the patterns of brain activity and connectivity measured using functional neuroimaging. Of note, a shared disruption in the functional connectivity among large-scale networks (frontoparietal network, somatomotor network, salience network, subcortical network) is presumed to exist across MPDs (Baker et al., 2019; Huang et al., 2020; Sharma et al., 2017; Xia et al., 2018). Various distributed networks have been implicated in specific symptom patterns (e.g. default mode network in psychosis, ventral attentional network, and salience networks in mood symptoms) (Huang et al., 2020; Pan et al., 2022; Xia et al., 2018). Nevertheless, it is unclear that if ‘dysconnectivity’ *per se* (i.e. a failure of coordinated brain activity) is the common pathophysiological substrate among the MPDs, or if such shared patterns emerge from multiple disruptions in local functional integrity (i.e. a failure of tonic regional brain function), or both.

The integrity of connections between two brain regions A and B could be influenced by both (1) the status of the pathway between A and B, and (2) the regional functional status of either A or B. In disease states, it is crucial to understand whether abnormal connectivity within a large-scale network arises due to regional aberrations or due to disturbances primarily in communication processes that bind regions together at a functional level across time. Such an understanding will not only help resolve the basis of complex dysconnectivity patterns, but also aid in choosing targets for neuromodulatory treatment approaches that are rising to prominence in recent times.

The “local” tonic integrity of a brain region can be studied using multiple measures obtained from resting-state fMRI: (1) the homogeneity or concordance of blood oxygen level dependent (BOLD) fMRI signal fluctuations with neighboring voxels measured using Regional Homogeneity (Reho) (Zang, Jiang, Lu, He, & Tian, 2004); (2) the amplitude of low frequency fluctuations (ALFF) in BOLD signals in the region, reflecting the magnitude of tonic regional BOLD signal (Zou et al., 2008); (3) the number of distributed connections made by a specific region relative to the rest of the brain, known as the normalized degree centrality (DC), reflecting the relative participation of a region in the low frequency signal variations of the rest of the brain (Buckner et al., 2009). Reho and ALFF explain a portion of variance in task related functional activations of brain regions (Yuan et al., 2013). DC from many brain regions relates linearly to the strength of their connectivity with other brain regions (Di et al., 2013). Further, localized metabolic activity appears to be tightly linked to regional centrality (Liang, Zou, He, & Yang, 2013; Lord, Expert, Huckins, & Turkheimer, 2013; Tomasi, Wang, & Volkow, 2013). Therefore, the above three indices, averaged over time from a resting-state fMRI acquisition, can be considered to reflect the state of tonic regional brain function or regional functional efficiency (RFE) (Di Martino et al., 2014). In contrast to the utilization of individual regional measures, RFE offers a more holistic and nuanced characterization of the regional function of a particular brain region.

Localized abnormalities in the RFE measures have been observed in several neuropsychiatric disorders including schizophrenia (He et al., 2013; Liu et al., 2016; Palaniyappan & Liddle, 2014) and in transdiagnostic studies (Wei et al., 2020) along with abnormalities in distributed brain connectivity (Pettersson-Yeo, Allen, Benetti, McGuire, & Mechelli, 2011). In schizophrenia, Zalesky et al. (Zalesky, Fornito, Egan, Pantelis, & Bullmore, 2012) demonstrated abnormal RFE (Reho and ALFF) in all regions with large-scale dysconnectivity in patients. Nevertheless, the relationship between these two sets of abnormalities (regional integrity and inter-regional connectivity) in other MPDs is not known. Further no previous studies have identified regions showing conjoint abnormalities in all three measures of RFE in MPDs to date. Such regions with low RFE may benefit form focal neuromodulation. On the other hand, concurrent targeting at multiple sites may be required to restore a network that is disconnected despite intact RFE of its constituent nodes.

Conventional estimates of derived variables from fMRI assumed “stationary” resting state relationships and ignored the temporal variability of brain over time. More recent studies have uncovered the highly dynamic non-stationary spatiotemporal functional organization, as reflected by dynamic variability in functional connectivity estimated across short time windows (Deco, Jirsa, & McIntosh, 2011; Hutchison et al., 2013). Aberrant dynamic activity and functional connectivity have been identified in MPDs, as evidenced by many studies (Luo et al., 2023; Tian et al., 2023; Zhou et al., 2021), including our previous work (Sun, Liu, Yang, Fan, & Yang, 2021; Wang et al., 2024; Yang et al., 2022). A recent study reported that disrupted variability in fMRI signal across the frontotemporal language network has been identified as a core common substrate across MPDs (Wei et al., 2023). Fluctuations across time can be computed for Reho, ALFF, as well as DC, with the quantified dynamic values indicating the degree of phasic variability in BOLD signal within a voxel (dALFF), neighboring voxels (dReho), or distant voxels (dDC). We consider the regions that show a combined diagnostic effect on all three static RFE measures to be prime suspects for tonic deficits in localized brain activity across MPDs, while those with a disruption restricted to the dynamic measures to be primarily “disconnected” in their temporal coordination with other areas, with minimal or no regional deficits *per se*. This characterization is based on previous works (Magnuson, Thompson, Pan, & Keilholz, 2014; Majeed et al., 2011; Matsui, Murakami, & Ohki, 2019; Thompson, 2018) (see the online Supplementary Material S1 for more details) and provides a nuanced understanding of the temporal dynamics underlying neural activity and connectivity.

In the present study using fMRI at rest, we aimed to identify the brain regions showing a conjoint abnormality in the three measures of RFE (static) and the corresponding dynamic measures in MPDs. We aimed to derive a comprehensive characterization of “tonic” resting activity (over minutes) and dynamic variability (over seconds), and their overlapping nature across SCZ, BD, and MDD. The potential associations between the observed patterns and both clinical symptomatology and cognitive impairment were further explored. We also sought to determine if these patterns are influenced by genetic substrates using an indirect (out-of-sample) approach. Gene annotation and enrichment analyses would facilitate the exploration of genes and genetic pathways associated with MPDs, providing insights into the intricate molecular pathways.

## Methods

### Participants

This study initially recruited 314 patients (124 with schizophrenia [SCZ], 88 with bipolar disorder [BD], and 102 with major depressive disorder [MDD]) from the Second Xiangya Hospital (Datasets #1 and #2), Central South University, and 80 healthy controls (HCs) from the community. Each participant was a right-handed native Chinese speaker and was provided written informed consent. This study was approved by the Ethics Committee of the Second Xiangya Hospital of Central South University. All procedures were conducted in strict accordance with the Declaration of Helsinki.

All the patients were diagnosed by board-certified psychiatrists using the DSM-5- criteria for SCZ, BD, and MDD. Patients aged 18~50 years old with at least 9 years of education were included. Patients were excluded if they met the following criteria: (1) metal devices such as electronic implants and any other contraindications to MRI; (2) have received electroconvulsive therapy; (3) presence of substance abuse or dependence and major physical illness; (4) history of neurological disorder. The other criteria for BD and MDD (with or without psychotic features), and states of BD are described in online Supplementary Material S2. The inclusion and exclusion criteria for HCs were the same as those for patients except that the HCs and their first-degree relatives did not have personal histories of any psychiatric disorders. All the participants completed clinical and cognitive assessments and resting-state fMRI on the same day.

### Clinical and cognitive assessments

The severity of psychotic symptoms was rated by the Brief Psychiatric Rating Scale (BPRS) in all the patients, and Positive and Negative Syndrome Scale (PANSS) in SCZ. The severity of mania and depression was evaluated using the Young Mania Rating Scale (YMRS) and the Hamilton Rating Scale for Depression (HAMD) in BD and MDD. In addition, the cognitive function was evaluated using the information of Wechsler Adult Intelligence Scale (WAIS_Information) and digit symbol subtests of Wechsler Adult Intelligence Scale (WAIS_Digit symbol) and N-back tests. These cognitive tests have been previously used by our research group in SCZ (Pan et al., 2020, 2022).

### ALFF, Reho, and DC calculation

The details of fMRI data imaging acquisition parameters and data preprocessing are described in online Supplementary Material S3 and S4. For static ALFF (sALFF), we transformed the time series of each voxel to frequency domain and calculated the square root at each frequency. The mean square root across frequency band 0.01–0.08 Hz for each voxel was obtained (Zang et al., 2007). For static Reho (sReho), the Kendall's coefficient of concordance was calculated between the BOLD time-series for each voxel and those of its 26 nearest neighbors (Zuo et al., 2013). For static DC (sDC), we performed correlations between the time-series of each voxel with every other voxel and restricted the correlations to positive correlations above a threshold of *r* = 0.25. The generated ALFF, Reho, and DC values were transferred to *z* values by the Fisher *z* transformation to achieve normality. Reho and DC maps were smoothed with FWHM = 8 mm.

The dynamic DC (dDC), dynamic Reho (dReho), and dALFF metrics were calculated using a sliding window approach via DynamicBC toolbox (Liao et al., 2014). According to previous studies, the minimum window length should be no less than 1/*f*_min_ to reduce spurious fluctuations caused by too short window length (Leonardi & Van De Ville, 2015). The *f*min means the minimum frequency of time series (Leonardi & Van De Ville, 2015). We adopted an empirically validated window length of 50 TRs as suggested by our previous studies (Sun et al., 2022; Yang et al., 2022). For Dataset #1, the full-length time series were comprised of 240 TRs (480s), and the windows were shifted by 1 TR (2s). The time series were then divided into 191 windows for each subject. For Dataset #2, the time series was comprised of 206 TRs (412s), and the window was shifted by 1 TR (2s). The full-length time series were then divided into 157 windows for each subject. We obtained the dDC, dReho, and dALFF maps for each sliding window. Similar to static metrics, we restricted dDC calculation to positive correlations above a threshold of *r* *=* 0.25, dReho with a cluster size of 27 voxels, and dALFF with the frequency band 0.01–0.08 Hz. The coefficients of variance of dDC, dReho, and dALFF across all sliding windows were computed. At last, the dReho and dDC were smoothed with FWHM = 8 mm.

### Principal component analysis

We stacked three static metrics (sALFF, sReho, and sDC) into a matrix, and normalized this matrix before conducted the principal component analysis (PCA). We extracted the first component to represent the synthesized static RFE. The same procedure was repeated for three dynamic metrics (dALFF, dReho, and dDC). The first component was deemed to dynamic RFE. The variances explained by the first component of static/dynamic RFE, and the loadings of the first component of static/dynamic RFE on each metric are provided in online Supplementary Tables S1, S2.

### Statistical analysis

We used the SPSS 22.0 (SPSS, Inc., Chicago, IL) to compare the demographic, clinical, and cognitive characteristics among groups. The one-way ANOVA or *t* test was used for continuous variables and χ^2^ test was used for categorical variables (*p* < 0.05). To compare the difference of static and dynamic RFE, one-way ANCOVA was performed across four groups with age, gender, education, site, and mean FD as covariates in SPM 8 program. The voxel-wise threshold of statistical significance was set at false discovery rate corrected *p* (*p*_FDR_) < 0.05 and cluster size>20 voxels. The voxels with significant differences in ANCOVA were masked for further post-hoc *t* tests (*p*_FDR_ < 0.05). We performed the Pearson correlation analyses to relate the clinical and cognitive characteristics with RFE abnormalities after age, gender, education, site, and mean FD controlled (uncorrected *p* < 0.05).

### Exploratory analysis

We undertook a second-level indirect exploration to understand the implications of observed RFE changes in terms of gene expression using extrinsic information (i.e. no direct genetic data from participants were used).

#### Gene and functional annotation analysis

We used the Brain Annotation Toolbox (BAT) (Liu et al., 2019) to perform functional and genetic annotation analysis on the observed regions with abnormal static or dynamic RFE. The BAT could deliver functional information from Neurosynth (Yarkoni, Poldrack, Nichols, Van Essen, & Wager, 2011) and gene expression profiles from the Allen Human Brain Atlas (AHBA) (Shen, Overly, & Jones, 2012) based on the brain regions consisting of clusters of voxels (Liu et al., 2019). In the functional annotation, the permutation analysis is performed to get a null distribution of the activation ratio for each functional term in the Neurosynth database; in the genetic annotation, the permutation analysis is performed to identify the differentially expressed genes in the given regions compared with samples in the background (Liu et al., 2019). The permutation times were set as 5000 times. The other parameters for genetic annotations were as follows: ROI size = 6 mm, minimal sample size = 5.

#### Enrichment analysis

We uploaded the derived differentially expressed genes to the Database for Annotation, Visualization, and Integrated Discovery (https://david.ncifcrf.gov/). The Gene Ontology (GO) database, specifically focusing on three domains including the biological process, cellular component, and molecular function, and Kyoto Encyclopedia of Genes and Genomes (KEGG) database for Homo sapiens sets were used to achieve gene function and pathway enrichment analysis. The statistical significance level was set as *p*_FDR_ < 0.05.

## Results

### Demographic and clinical characteristics

A total of 364 participants (118 patients with SCZ, 80 patients with BD, 91 patients with MDD, and 75 HCs) were enrolled for this study. The demographic and clinical characteristics are shown in Table 1. As expected, there were significant differences in gender, age, and education years across four groups. Significant differences in duration, medication, and BPRS were noted among patient groups. SCZ showed the lowest WAIS_Information and WAIS_Digit symbol scores and the poorest N-back test performances among four groups. Details of characteristics for the two datasets are provided in online Supplementary Tables S3, S4. There were 32 patients with MDD and psychotic features and 25 patients with BD and psychotic features. In BD, there were 36 patients in a depressive state, 15 in a manic/hypomanic state, 1 in a mixed state, and 28 in a euthymic state.

**Table 1.** Demographic, clinical, and cognitive characteristics of each group

Variables	SCZ (*n* = 118)	BD (*n* = 80)	MDD (*n* = 91)	HCs (*n* = 75)	χ^2^/*F*/*t*	*p*	Post hoc analysis^a^		
							Comparisons	χ^2^/*LSD-t*	*p*
Gender (M/F)	79/39	37/43	42/49	34/41	14.15^b^	0.003	SCZ-BD	8.42	0.004
							SCZ-MDD	9.11	0.003
							SCZ-HCs	8.83	0.003
Age (yr)	24.3 ± 5.6	27.4 ± 7.5	27.6 ± 8.3	23.9 ± 5.4	7.65^c^	<0.001	SCZ < BD	−3.20	0.001
							SCZ < MDD	−3.58	<0.001
							BD > HCs	3.19	0.002
							MDD > HCs	3.52	<0.001
Education (yr)	11.8 ± 2.6	13.1 ± 2.9	13.1 ± 2.7	14.1 ± 2.3	12.40^c^	<0.001	SCZ < BD	−3.45	0.001
							SCZ < MDD	−3.49	0.001
							SCZ < HCs	−5.92	<0.001
							BD < HCs	−2.34	0.020
							MDD < HCs	−2.49	0.013
Illness duration (m)	27.9 ± 33.0	60.8 ± 65.5	40.8 ± 56.8	–	9.78^c^	<0.001	SCZ < BD	−4.42	<0.001
							BD > MDD	2.53	0.012
CPZ	434.8 ± 218.6	226.1 ± 246.5	14.7 ± 58.4	–	121.70^c^	<0.001	SCZ > BD	6.81	<0.001
							SCZ > MDD	10.81	<0.001
							BD > MDD	4.97	<0.001
FLU	0.7 ± 3.8	11.3 ± 14.7	16.4 ± 20.3	–	34.44^c^	<0.001	SCZ < BD	−6.09	<0.001
							SCZ < MDD	−12.78	<0.001
							BD < MDD	−7.38	<0.001
PANSS-P	15.15 ± 5.65	–	–	–	–	–	–	–	–
PANSS-N	17.72 ± 9.06	–	–	–	–	–	–	–	–
PANSS-G	31.05 ± 10.46	–	–	–	–	–	–	–	–
BPRS	36.6 ± 10.8	26.4 ± 7.1	32.4 ± 7.0	–	26.53^c^	<0.001	SCZ > BD	7.13	<0.001
							SCZ > MDD	4.40	<0.001
YMRS	–	5.8 ± 8.5	2.3 ± 2.5	–	3.32^d^	0.011	–	–	–
HAMD	–	12.7 ± 9.4	21.5 ± 4.3	–	−7.59^d^	<0.001	–	–	–
WAIS_Information	16.3 ± 4.8	19.5 ± 4.6	17.9 ± 5.9	20.8 ± 4.6	10.11^c^	<0.001	SCZ < BD	−3.73	<0.001
							SCZ < HCs	−5.30	<0.001
							MDD < HCs	−3.20	0.002
WAIS_Digit symbol	65.6 ± 15.8	67.4 ± 16.9	68.5 ± 19.2	89.5 ± 12.8	34.60^c^	<0.001	SCZ < HCs	−8.54	<0.001
							BD < HCs	−8.52	<0.001
							MDD < HCs	−6.69	<0.001
0-back target ACC	0.8 ± 0.3	0.9 ± 0.2	0.9 ± 0.2	0.9 ± 0.1	8.37^c^	<0.001	SCZ < BD	−3.08	0.002
							SCZ < MDD	−3.03	0.003
							SCZ < HCs	−4.62	<0.001
0-back target RT	556.7 ± 135.9	548.9 ± 125.7	545.4 ± 110.9	485.0 ± 88.0	5.59^c^	0.001	SCZ > HCs	3.85	<0.001
							BD > HCs	3.03	0.003
							MDD > HCs	2.41	0.017
2-back target ACC	0.5 ± 0.3	0.6 ± 0.3	0.7 ± 0.2	0.7 ± 0.2	14.48^c^	<0.001	SCZ < BD	−2.53	0.012
							SCZ < MDD	−3.12	0.002
							SCZ < HCs	−6.51	<0.001
							BD < HCs	−3.32	0.001
2-back target RT	707.6 ± 188.9	744.2 ± 213.5	717.1 ± 176.5	650.4 ± 194.6	5.00^c^	0.002	SCZ > HCs	2.88	0.004
							BD > HCs	3.59	<0.001
							MDD > HCs	2.36	0.019

Abbreviations: SCZ, Schizophrenia; BD, bipolar disorder; MDD, major depressive disorder patients; HCs, healthy controls; M/F, male/female; yr, year; m, month; CPZ, chlorpromazine equivalent dose (Leucht et al., 2015); FLU, fluoxetine equivalent dose (Hayasaka et al., 2015); PANSS-P, N, G, positive, negative, general psychopathology subscale in Positive and Negative Syndrome Scale; BPRS, Brief Psychiatric Rating Scale; YMRS, Young Mania Rating Scale; HAMD, Hamilton Depression Rating Scale; ACC, accuracy; RT, response time.

a This table only showed the significant results in post hoc analysis. We performed χ^2^ test for gender and *t* test for other variables in post hoc analysis.

b χ^2^ test.

c One-way ANOVA.

d Two-sample *t* test.

*Note*: Quantitative data were presented as mean ± standard deviation.

### Group differences in static and dynamic RFE

**Figure 1.**
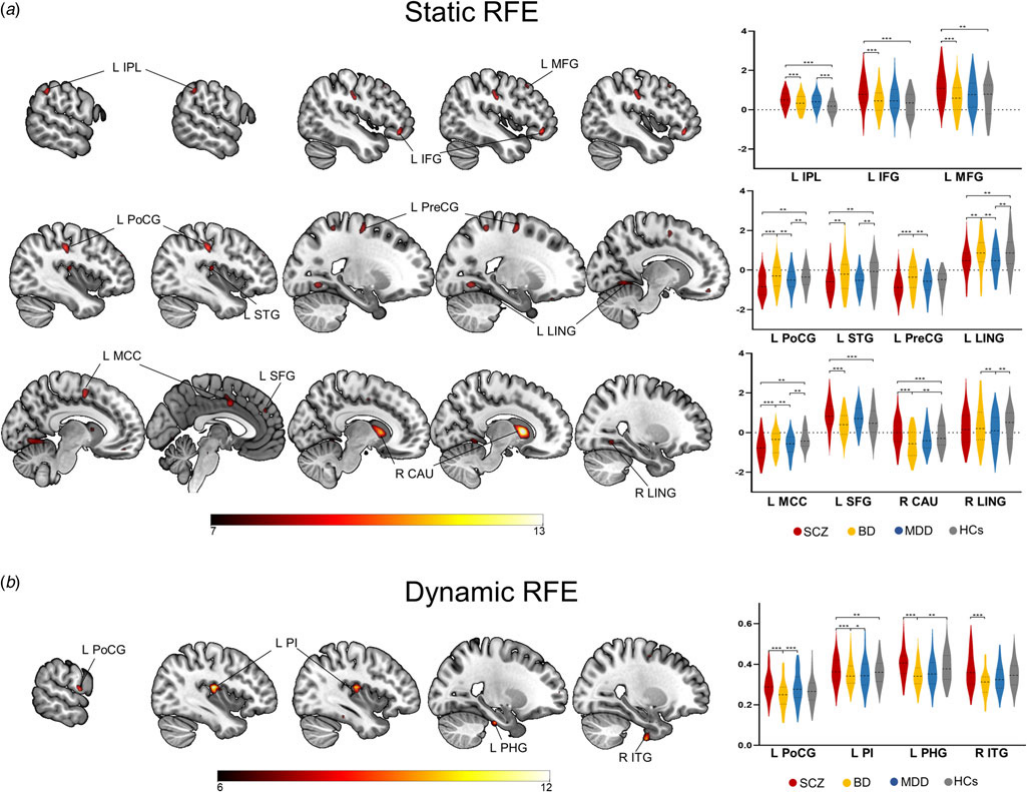
Significant differences in static and dynamic RFE among all groups. Brain maps depict the clusters with omnibus differences in static and dynamic RFE, and violin plots show the results in post hoc comparisons. SCZ, schizophrenia; BD, bipolar disorder; MDD, major depressive disorder patients; HCs, healthy controls; L, left; R, right; IPL, inferior parietal lobule; IFG, inferior frontal gyrus; MFG, middle frontal gyrus; PoCG, postcentral gyrus; STG, superior temporal gyrus; PreCG, precentral gyrus; LING, lingual gyrus; MCC, middle cingulate cortex; SFG, superior frontal gyrus; CAU, caudate; PI, posterior insula; PHG, parahippocampal gyrus; ITG, inferior temporal gyrus. * *p_FDR_* < 0.05; ** *p_FDR_* < 0.01; *** *p_FDR_* < 0.001.

As shown in Fig. 1a and Table 2, we observed the significant differences across four groups in static RFE in the right caudate (*η*^2^ = 0.11), left postcentral gyrus (*η*^2^ = 0.08), left inferior frontal gyrus (IFG; *η*^2^ = 0.07), left inferior parietal lobule (IPL; *η*^2^ = 0.07), left superior temporal gyrus (STG; *η*^2^ = 0.07), left middle cingulate cortex (MCC; *η*^2^ = 0.07), left middle frontal gyrus (MFG; *η*^2^ = 0.07), bilateral lingual gyrus (left: *η*^2^ = 0.07; right: *η*^2^ = 0.06), left precentral gyrus (*η*^2^ = 0.07), and left superior frontal gyrus (SFG; *η*^2^ = 0.06). Post hoc tests revealed that most regions showed aberrant static RFE in SCZ compared to BD and HCs.

**Table 2.** Significant differences in static and dynamic RFE among all groups

Regions	One-way ANCOVA							Post-hoc analysis			
	*k*	MNI			*F*_3, 360_	*p*_FDR_	*h*^2^	Comparison	*t*	*p*_FDR_	Cohen's *d*
		*X*	*Y*	*Z*							
Static RFE											
R CAU	126	12	12	6	14.17	<0.001	0.11	SCZ > BD	6.46	<0.001	0.94
								SCZ > HCs	4.74	<0.001	0.71
								BD < HCs	−4.21	0.005	0.68
L PoCG	120	−39	−24	39	10.29	0.004	0.08	SCZ < BD	−5.11	<0.001	0.74
								SCZ < HCs	−3.87	0.001	0.58
								BD > MDD	3.53	0.003	0.54
								MDD < HCs	−3.82	0.001	0.60
L IFG	51	−45	39	−9	9.52	0.007	0.07	SCZ > BD	3.91	<0.001	0.57
								SCZ > HCs	5.24	<0.001	0.78
L IPL	22	−60	−45	45	9.50	0.007	0.07	SCZ > BD	3.92	<0.001	0.57
								SCZ > HCs	4.93	<0.001	0.74
								MDD > HCs	5.02	<0.001	0.79
L STG	42	−39	−18	15	9.07	0.008	0.07	SCZ < BD	−3.47	0.002	0.51
								SCZ < HCs	−4.61	0.001	0.69
								MDD < HCs	−4.30	0.001	0.68
L MCC	66	−9	−3	51	8.91	0.010	0.07	SCZ < BD	−4.78	<0.001	0.70
								SCZ < HCs	−4.54	0.001	0.68
								BD > MDD	3.16	0.006	0.49
								MDD < HCs	−3.76	0.001	0.59
L MFG	21	−45	18	48	8.64	0.012	0.07	SCZ > BD	3.94	<0.001	0.57
								SCZ > HCs	3.9	0.001	0.58
L LING	115	−12	−60	−9	8.61	0.012	0.07	SCZ < BD	−3.83	0.001	0.56
								SCZ < HCs	−4.15	0.001	0.62
								BD > MDD	4.07	0.003	0.63
								MDD < HCs	−4.34	0.001	0.68
L PreCG	24	−21	−12	60	8.56	0.012	0.07	SCZ < BD	−4.60	<0.001	0.67
								BD > MDD	4.08	0.003	0.63
R LING	51	27	−57	−9	8.21	0.013	0.06	BD > MDD	4.08	0.003	0.63
								MDD < HCs	−4.63	0.001	0.73
L SFG	20	−3	45	33	8.14	0.014	0.06	SCZ > BD	4.51	<0.001	0.66
								SCZ > HCs	3.98	<0.001	0.59
Dynamic RFE											
L PI	33	−39	−18	−15	12.86	0.015	0.10	SCZ > BD	5.72	<0.001	0.83
								SCZ > HCs	4.08	0.002	0.61
								BD < MDD	−3.08	0.012	0.47
L PHG	29	−24	−21	−30	10.69	0.033	0.08	SCZ > BD	4.92	<0.001	0.72
								BD < HCs	−4.25	0.001	0.69
R ITG	30	27	−9	−51	10.65	0.033	0.08	SCZ > BD	5.19	<0.001	0.76
L PoCG	25	−63	−6	12	9.93	0.045	0.08	SCZ > BD	3.97	<0.001	0.58
								BD < MDD	−4.89	<0.001	0.75

Abbreviations: FDR, false discovery rate correction; SCZ, schizophrenia; BD, bipolar disorder; MDD, major depressive disorder patients; HCs, healthy controls; k, cluster extension in number of voxels; MNI, Montreal Neurological Institute; L, left; R, right; CAU, caudate; PoCG, postcentral gyrus; IFG, inferior frontal gyrus; IPL, inferior parietal lobe; STG, superior temporal gyrus; MCC, middle cingulate cortex; MFG, middle frontal gyrus; LING, lingual gyrus; PreCG, precentral gyrus; SFG, superior frontal gyrus; PI, posterior insula; PHG, parahippocampal gyrus; ITG, inferior temporal gyrus.

As shown in Fig. 1b and Table 2, the dynamic RFE in the left posterior insula (PI; *η*^2^ = 0.10), left parahippocampal gyrus (*η*^2^ = 0.08), right inferior temporal gyrus (ITG; *η*^2^ = 0.08), and left postcentral gyrus (*η*^2^ = 0.08) showed significant differences across four groups. All regions showed higher dynamic RFE in SCZ compared to BD.

Additionally, we compared the static and dynamic RFE among SCZ, BD without psychotic features, MDD without psychotic features, and HCs. The comparison results (online Supplementary Table S5) were consistent with the main results. We also compared the static and dynamic RFE among SCZ, BD without and without psychotic features/BD in depressive and non-depressive states, MDD, and HCs (online Supplementary Fig. S1).

### Correlation of RFE abnormalities with clinical and cognitive characteristics

**Figure 2.**
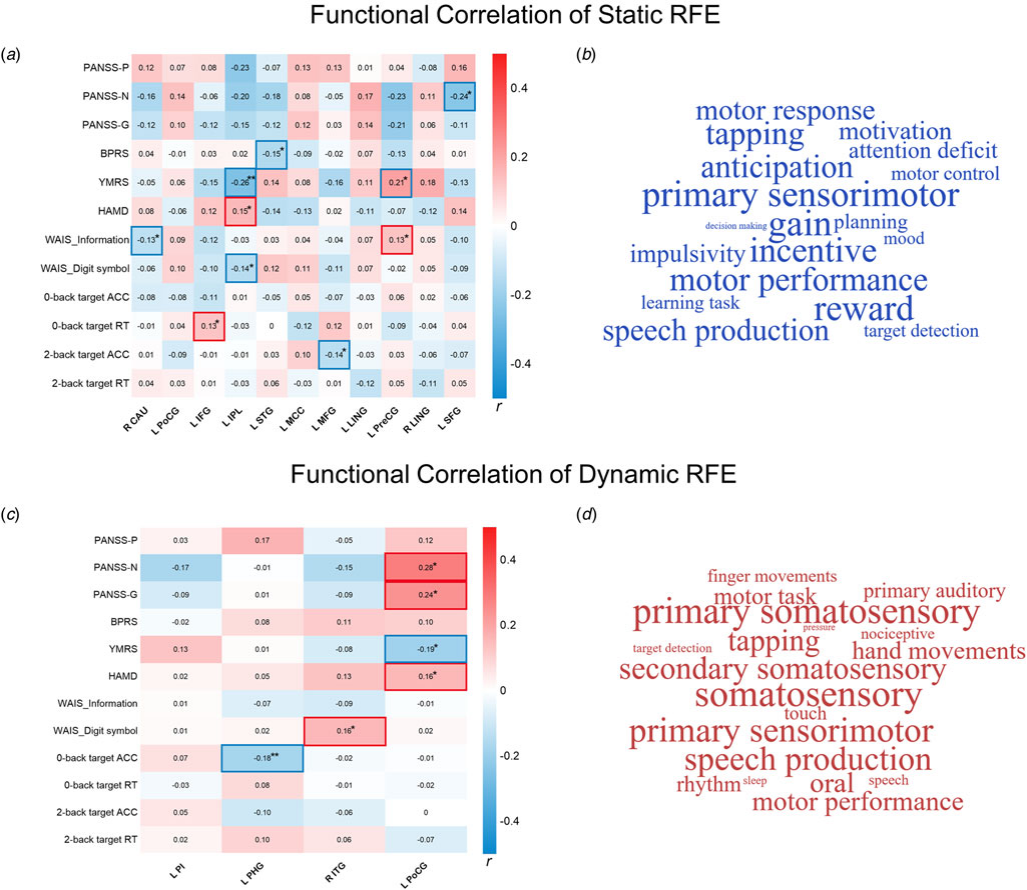
Functional correlation of transdiagnostic static and dynamic RFE abnormalities. Heat maps (a and c) depict the correlation between clusters with omnibus differences in static and dynamic RFE and clinical and cognitive characteristics respectively. Word cloud maps (b and d) show the functional terms related to overall static and dynamic RFE via Neurosynth respectively. *Note*: PANSS was only used in SCZ; YMRS and HAMD were used in BD and MDD. PANSS-P, N, and G, positive, negative, and general psychopathology subscale in Positive and Negative Syndrome Scale; BPRS, Brief Psychiatric Rating Scale; YMRS, Young Mania Rating Scale; HAMD, Hamilton Depression Rating Scale; L, left; R, right; CAU, caudate; PoCG, postcentral gyrus; IFG, inferior frontal gyrus; IPL, inferior parietal lobule; STG, superior temporal gyrus; MCC, middle cingulate cortex; MFG, middle frontal gyrus; LING, lingual gyrus; PreCG, precentral gyrus; SFG, superior frontal gyrus; PI, posterior insula; PHG, parahippocampal gyrus; ITG, inferior temporal gyrus.* *p* < 0.05; ** *p* < 0.01; *** *p* < 0.001.

For the static RFE, we found a negative correlation between static RFE in the left SFG and PANSS negative subscale scores (*r* = −0.24) and between static RFE in the left STG and BPRS scores (*r* = −0.15). The static RFE in the left IPL was related to HAMD (*r* = 0.15) and YMRS (*r* = −0.26), and WAIS_Digit symbol (*r* = −0.14). The static RFE in the left precentral gyrus was related to the YMRS scores (*r* = 0.21) and WAIS_Information (*r* = 0.13). The details of correlation of certain regions with abnormal static RFE are shown in Fig. 2a. The functional annotation revealed close association between overall static RFE abnormalities and sensorimotor and reward functions across groups (*p* < 0.05; Fig. 2b).

For the dynamic RFE, we observed that the dynamic RFE in the left postcentral gyrus was positively associated with PANSS negative (*r* = 0.28) and general psychopathology (*r* = 0.24) subscales, HAMD (*r* = 0.16), and negatively with YMRS (*r* = −0.19). The dynamic RFE in the left parahippocampal gyrus was related to 0-back target accuracy (*r* = −0.18). The details of correlation of dynamic RFE are shown in Fig. 2c. The functional annotation suggested overall dynamic RFE abnormalities were related to somatosensory and sensorimotor functions across groups (*p* < 0.05; Fig. 2d).

The correlation analyses were also performed to relate the illness duration with RFE abnormalities. We only found static RFE in left IFG was negatively related to the illness duration (*r* *=* −0.15, *p* *=* 0.015; online Supplementary Table S6).

### Exploratory analysis

**Figure 3.**
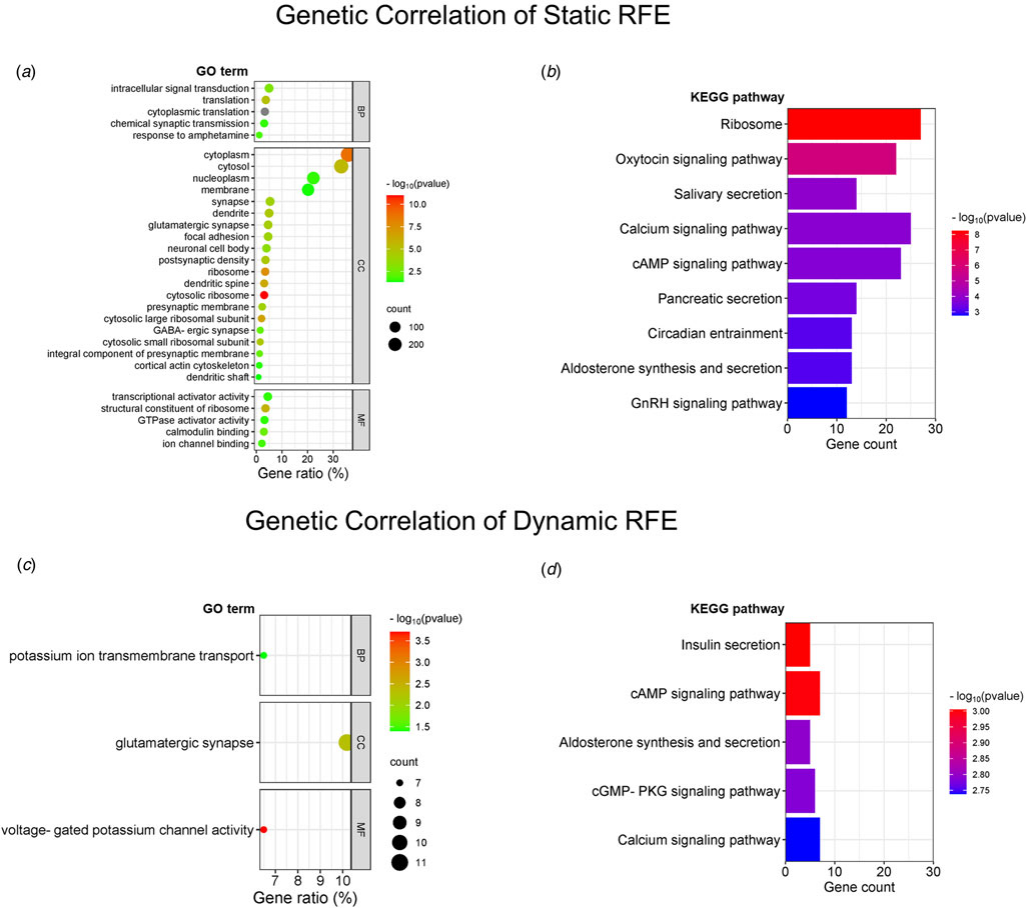
Genetic correlation of static and dynamic RFE abnormalities. The enrichment results are arranged by GO terms (a and c) and KEGG pathways (b and d). BP, biological process; CC, cellular component; MF, molecular function.

The genetic annotation analyses identified 994 and 114 statistically differentially overexpressed genes for abnormal regions of static and dynamic RFE respectively (*p*_FDR_ < 0.05). These genes mainly enriched in GO terms of cellular components for both static and dynamic RFE (all *p*_FDR_ < 0.05; Fig. 3a and 3c), especially ‘cytoplasm’ for static RFE and ‘glutamatergic synapse’ for dynamic RFE. Potassium transport and voltage gated potassium channel activity was also related to dynamic RFE changes. The KEGG pathway enrichment analyses revealed that differentially expressed genes were enriched in calcium/cAMP signaling for static and dynamic RFE (all *p*_FDR_ < 0.05; Fig. 3b and 3d).

## Discussion

The present study revealed the dysfunctional patterns in the static and dynamic RFE across SCZ, BD, and MDD, with a potential link to differentially gene expression profiles and signaling pathways. The graded alterations in static and dynamic RFE in MPDs may bridge diagnostic categories, concordant with clinical symptoms and cognitive dysfunctions. We report four main findings here. First, SCZ showed prominently higher static RFE in subcortical regions and some of the frontal and parietal cortices than BD and HCs. SCZ and MDD exhibited lower static RFE in sensorimotor regions compared to BD; this reduction relates mostly to depressive and negative symptom burden. Second, SCZ showed generally higher dynamic RFE in the cortico-limbic regions than BD. Third, functional annotation analysis suggested a potential association between overall static RFE abnormalities and reward and sensorimotor functions and between overall dynamic RFE abnormalities and sensorimotor functions. Fourth, gene annotation and enrichment analysis suggested that differentially overexpressed genes associated with abnormal regions may be involved in “glutamatergic synapse” and calcium/cAMP signaling for static/dynamic RFE and “potassium ion channel” for dynamic RFE.

We observed that higher static RFE in caudate was shown in SCZ compared to BD and HCs, and in HCs compared to BD. The caudate is a key striatal component of the reward circuit mediating processing emotional and motivational information (Haber & Knutson, 2010), align with the association of reward function indicated by our functional annotation analysis. The striatum acts as an integrative hub that receives inputs from multiple distal cortical regions and via various connections with the midbrain (McCutcheon, Abi-Dargham, & Howes, 2019). Combining our results of higher static RFE in frontoparietal regions and lower static RFE in sensorimotor regions in SCZ, we assumed that integration of cortical inputs from emotional, cognitive, and motor areas disrupted in SCZ, which may be explained by disorganized dopamine signaling (Horga et al., 2016). It is known that dysregulated dopaminergic modulation of striatal function contributes to the symptoms of schizophrenia (Lieberman & First, 2018). Neuroimaging studies have demonstrated reduced striatal activation during reward anticipation in patients with psychosis (Radua et al., 2015), which may be associated with disrupted striatal dopamine release in response to reward-indicating cues (Maia & Frank, 2017). A PET study found that the dopamine synthesis capacity in the dorsal caudate progressively increased with the development of psychosis (Howes et al., 2011). Consistent with previous findings of disrupted striatal activity and corticostriatal connectivity (Fornito et al., 2013; Zhao et al., 2018), our results highlighted the diminished tonic regional function of caudate in SCZ from a transdiagnostic perspective. Whether the altered striatal dopamine release to neural activation will be a distinct mechanism for the diminished tonic regional function of caudate in SCZ requires further exploration across diagnostic categories.

The sensorimotor regions, including the precentral gyrus and postcentral gyrus, showed pronounced lower static RFE and higher dynamic RFE in SCZ and MDD compared with BD, indicating inefficiency and instability of sensorimotor regions in SCZ and MDD. In line with our results, previous meta-analyses found that decreased sALFF, sReho, and grey matter volume in the precentral gyrus and postcentral gyrus was shown in SCZ, but not in BD, relative to HCs (Gong et al., 2020; Qi et al., 2022; Vargas, López-Jaramillo, & Vieta, 2013). A finding supported by the eigenvector centrality mapping suggested that SCZ showed decreased global connectivity and centrality in somatosensory regions than BD and HCs (Skåtun et al., 2016). MDD exhibited significantly higher temporal stability in a state characterized by weak functional connectivity within and between the somatosensory motor network and relatively strong averaged functional activity of regions located in the somatosensory motor, salience, and dorsal attention networks (Javaheripour et al., 2023). The functional annotation analysis suggested potential association between overall static and dynamic RFE abnormalities and sensorimotor function. Our correlation analyses also reported that located in the sensorimotor regions, higher static activity and connectivity may be related to increased severity of manic symptoms, and lower dynamic activity and connectivity may be related to increased severity of negative and general symptoms, depressive symptoms, and decreased severity of manic symptoms. We assumed that the disrupted sensorimotor system may underlie the psychomotor dysfunctions and negative symptoms in SCZ (Berman et al., 2016; Magioncalda et al., 2020), as well as psychomotor retardation in MDD (Xia et al., 2023).

We also found SCZ showed higher dynamic RFE than BD and HCs, indicating excessive variability in SCZ. It was known that the insula is the first cortical target of ascending intersensory and visceral sensory inputs, which plays a role in the integration of subjective sensations to guide decision making (Singer, Critchley, & Preuschoff, 2009). PI communicates with multiple regions like sensorimotor areas and track interoceptive signals from subcortical regions to the salience network (Uddin, 2015). A previous study found that SCZ and individuals with a high risk for psychosis showed hypoconnectivity between PI and somatosensory areas than healthy controls (Li et al., 2019). Our result complements the unstable function of the PI from the dynamic perspective. Combining with our results of disrupted RFE in sensorimotor areas in SCZ, we assumed that abnormal temporal variability of functional connectivity between PI and sensorimotor areas may lead to altered interoception, which has implications for specific psychotic symptoms (Yao & Thakkar, 2022).

Notably, the gene enrichment analysis revealed that regions with abnormal static and dynamic RFE both correlated to the GO term “glutamatergic synapse.” Glutamate belongs to excitatory neurotransmitters and glutamatergic dysfunction has been proposed as an etiology of SCZ (Kruse & Bustillo, 2022). Genetic evidence from a recent whole-exome sequencing study of a large sample suggested that three genes were clearly related to glutamatergic function for schizophrenia (Singh et al., 2022). The glutamatergic projection derives from widespread cortices to the striatum (Reubi & Cuenod, 1979). Our fMRI results showed that SCZ showed pronounced altered static in the frontal and parietal cortices and left caudate, and dynamic function in the temporal cortex. The disturbances in glutamate-mediated neurotransmission may play a potential role in the cortico-striatal circuit in SCZ pathogenesis. Intriguingly, we also observed abnormal static and dynamic RFE may link to ion channel, especially potassium ion channel. The potassium ion channel responds to voltage gating and calcium transport (Alam, Svalastoga, Martinez, Glennon, & Haavik, 2023), which was also indicated by the potential association of GO term “voltage-gated potassium channel activity” and KEGG pathway “calcium signaling pathway”. Growing evidence from genetic, animal, and preclinical studies have highlighted the potential therapeutic role of potassium ion channel in SCZ (Musselman et al., 2023), MDD (Costi, Han, & Murrough, 2022), and other neuropsychiatric disorders (Alam et al., 2023). The potassium ion channel may be a new target for understanding and treating MPDs (Alam et al., 2023).

This study has several limitations that should be noted. First, we found significant differences in gender, age, and education years across four groups. Although these demographic data were included as covariates, non-linear confounding effects cannot be ruled out. Second, the sample size of BD in different states were not balanced. There were more patients in depressed state than in manic/hypomanic state and mixed state. Besides, nearly one-third of BD and MDD had psychotic features. The common psychotic features may contribute to shared neural dysfunction in SCZ and MDD, though this may not explain all the shared variance in the current sample. Third, the medication exposure and illness duration may moderate brain activity and functional connectivity. Future longitudinal studies will be needed to unravel the effects of medication and illness duration on brain function. Fourth, our results of Pearson correlation analysis failed to survive FDR correction, so these results should be interpreted with caution. In addition, we did not validate our results using other independent transdiagnostic datasets.

In conclusion, RFE emerges as a potential transdiagnostic biomarker of MPDs with a substantial amount of its spatial distribution explained by genetic profiles. Among MPDs, SCZ, and MDD share more RFE patterns in common, but differ from BD, indicating the intrinsic link between depression and schizophrenia. These results provide novel insights into specific neurobiological mechanisms underpinning clinical symptoms of severe mental illnesses.

## Supporting information

Yang et al. supplementary material Yang et al. supplementary material

## Supplementary material

The supplementary material for this article can be found at https://doi.org/10.1017/S0033291724001685
