# Supplementary material for: Regional neural functional efficiency across schizophrenia, bipolar disorder, and major depressive disorder: a transdiagnostic resting-state fMRI study
Source: Psychol Med. 2024 Nov 18;54(15):4083–94. doi: 10.1017/S0033291724001685 (PMC11650196; doi:10.1017/S0033291724001685)
Supplement: Yang et al. supplementary material [file S0033291724001685sup001.docx]

**Supplementary Materials**

**S1 Detailed information about static and dynamic fMRI metrics**

Within the dynamic system of connected networks within the brain, voxel-based metrics of the BOLD signal averaged over a protracted time period i.e., ALFF, DC and ReHo are considered to be reflective of local brain properties of spatially discrete regions, independent of long-range connectivity (Li, Kadivar, Pluta, Dunlop, & Wang, 2012; Li, Zhu, Childress, Detre, & Wang, 2012; Lv et al., 2018). As we discussed in the manuscript, ALFF indexes low-frequency oscillatory contributions to BOLD signal in a local area. Reho indexes local connectivity in a discrete voxel by voxel neighborhood. DC, while a distributed connectivity-based metric, indexes the numerosity of connections (i.e., above-threshold correlations) that a single voxel makes with rest of the brain over a period; as such, the value of DC is more sensitive to local brain properties than to distant pathologies, and less sensitive to any single network-level disruption. This explains its robust correlation with ALFF (Sato et al., 2019). The tight link between these voxels based physiological properties and local glucose metabolism has been demonstrated in combined fMRI-PET studies in humans (Deng et al., 2022). Thus, disrupted integrity of the grey matter region containing the voxel of interest will be reflected in changes in ALFF, DC and ReHo (static, averaged over time). On the other hand, the functional implications of dynamic resting fMRI measures have been less well studied. These issues are succinctly summarized in an adversarial collaboration (Lurie et al., 2020) and by Thompson (Thompson, 2018).

We wish to highlight two lines of evidence that lend support for dynamic or time-varying BOLD-based measures to be physiological effects of distributed connectivity more than being local brain properties. Firstly, BOLD fluctuations propagate temporally over brain areas, contributing to the time-varying dynamic changes in voxel-based physiological properties (Majeed et al., 2011). These quasi-periodic patterns are disrupted when underlying structural connections are severed, as in the case of callosotomy, that gives rise to loss of coordinated fluctuations while the localized BOLD signal pattern over time is still recoverable (Magnuson, Thompson, Pan, & Keilholz, 2014). Secondly, simultaneous calcium imaging and optical hemodynamics indicate that the dynamic, time-varying fluctuations in BOLD are related to transient neural calcium potentials; on the other hand, these calcium transients produce a pattern that is inconsistent with stationary static connectivity, indicating that temporally sparse events indicating physiological coordination of action potentials across distributed regions, underwrite the dynamic functional measures (Matsui, Murakami, & Ohki, 2019).

Taken together, and as summarized by Thompson et al, in Fig 4 of their review (Thompson, 2018), as we move to more regional and more temporally resolved measures, the determinants of BOLD signal variations appear more reliant on coordinated communications and physiological fluctuations. These observations suggest that when only dynamic regional functional efficiency measures are disrupted, but when static RFE is intact, we can infer a failure of distributed temporal coordination that often occurs at a global scale. If a disruption of the tonic averages across time (static RFE) also occurs at the voxel level, this is more indicative of a process disrupting the physiological integrity of the observed voxel’s neighborhood. While this characterization provides a nuanced understanding of the temporal dynamics underlying neural activity and connectivity, we highlight to the readers that this is an assumption that helps in interpretation, based on circumstantial evidence as above, but still with no direct experimental evidence.

**S2 Supplementary criteria for BD and MDD with psychotic features and states of BD**

The patients with BD or MDD met DSM-5 criteria for BD with or without psychotic features, MDD with or without psychotic features respectively. BD and MDD with psychotic features were also required to have a minimum total score of 5 on four items from the Brief Psychotic Rating Scale (BPRS), including conceptual disorganization, suspiciousness, hallucinations, and unusual thought content (Keller et al., 2017).

In BD, consistent with our prior study (J. Yang et al., 2023), patients whose HAMD ≥17 and YMRS score <12 were considered depressive, patients whose HAMD <17 and YMRS score ≥12 were considered manic/hypomanic, patients whose HAMD ≥17 and YMRS score ≥12 were considered mixed, and patients whose HAMD score <17 and YMRS score <12 were considered euthymic.

**S3 fMRI data acquisition parameters**

Dataset #1: BOLD fMRI data were acquired on a Philips 3-T scanner with the following parameters: volumes = 250, matrix size = 64 × 64, slices number = 36, slices thickness = 4 mm, flip angle = 90°, FOV = 240 × 240 mm^2^, TR = 2000 ms, and TE = 30 ms.

Dataset #2: BOLD fMRI data were acquired on a Siemens Achieva 3-T scanner with the following parameters: volumes = 216, matrix = 64 × 64, slices number = 32, slice thickness = 4 mm, flip angle = 90°, FOV = 240 × 240 mm^2^, TR = 2000 ms, and TE = 30 ms.

**S4** **fMRI data preprocessing**

Data preprocessing was performed using the DPABI toolbox (Yan, Wang, Zuo, & Zang, 2016). We discarded the first 10 images to adjust for magnetic saturation delay. The remaining volumes were obtained for further preprocessing steps: slice timing correction, head motion realignment, spatial normalization with the brain template of Montreal Neurologic Institute (MNI). We performed smoothing (FWHM = 8mm) for subsequent DC and ALFF calculation but not for the Reho calculation. Nuisance covariates including 12 head motion parameters, white matter, ventricle signals, and temporal derivatives were regressed out. The global signal was retained because a recent study suggested that the global signal might carry illness-related information (G. J. Yang et al., 2014). Temporal band-pass filtering was performed between 0.01-0.08Hz to reduce low-frequency drift and high-frequency physiological noise for subsequent DC and Reho calculation but not for the ALFF calculation. The parameters from scrubbing data were also regressed out. Displaced volumes (framewise displacement [FD] > 0.5 mm) were interpolated by nearest-neighbor interpolation (Power, Barnes, Snyder, Schlaggar, & Petersen, 2012). Participants with head motion more than 2.5mm translation or 2.5° rotation in any direction and fMRI data failed to normalize to MNI space were excluded. A total of 364 participants (118 patients with SCZ, 80 with BD, 91 with MDD, 75 HCs) were included in the final analysis. We observed no significant differences in the mean FD among groups: SCZ, mean (SD) = 0.159 (0.067); BD, mean (SD) = 0.137 (0.066); MDD, mean (SD) = 0.142 (0.066); HCs, mean (SD) = 0.145 (0.050); F_3, 360_ = 2.40, *p* = 0.068.

**Table S1** The variances explained by the first component of dynamic RFE

| **Groups** | **Variances explained by the first component (%)** | |
| --- | --- | --- |
|  | **Static RFE** | **Dynamic RFE** |
| SCZ | 57.32 (5.54) | 93.60 (1.54) |
| BD | 58.82(4.38) | 93.56 (1.46) |
| MDD | 59.51(4.85) | 93.14 (1.48) |
| HCs | 56.67 (5.36) | 93.70 (1.35) |

Abbreviations: SCZ, Schizophrenia; BD, bipolar disorder; MDD, major depressive disorder patients; HCs, healthy controls.

**Table S2** The loadings of the first component of dynamic RFE on each brain metric

| **Groups** | **Loadings of the first component of**  **static RFE** | | |  | **Loadings of the first component of dynamic RFE** | | |
| --- | --- | --- | --- | --- | --- | --- | --- |
|  | **ALFF** | **Reho** | **DC** |  | **ALFF** | **Reho** | **DC** |
| SCZ | 0.44 (0.048) | 0.59 (0.150) | 0.64 (0.150) |  | 0.53 (0.037) | 0.65 (0.055) | 0.54 (0.038) |
| BD | 0.54 (0.036) | 0.64 (0.520) | 0.55 (0.033) |  | 0.54 (0.036) | 0.64 (0.052) | 0.55 (0.033) |
| MDD | 0.45 (0.040) | 0.60 (0.090) | 0.66 (0.052) |  | 0.50 (0.069) | 0.64 (0.068) | 0.57 (0.051) |
| HCs | 0.45 (0.040) | 0.58 (0.110) | 0.66 (0.054) |  | 0.52 (0.037) | 0.66 (0.052) | 0.53 (0.034) |

Abbreviations: SCZ, Schizophrenia; BD, bipolar disorder; MDD, major depressive disorder patients; HCs, healthy controls.**Table S3** Demographic, clinical, and cognitive characteristics of each group from Dataset #1

| Variables | SCZ (n=118) | BD (n=80) | MDD (n=43) | HCs (n=75) | *χ*^2^/*F*/*t* | *p* |
| --- | --- | --- | --- | --- | --- | --- |
| Gender (Male/Female) | 79/39 | 37/43 | 23/20 | 34/41 | 12.13 ^a^ | 0.007 |
| Age (years) | 24.25±5.60 | 27.39±7.53 | 31.70±8.96 | 23.92±5.39 | 17.01 ^b^ | <0.001 |
| Education (years) | 11.78±2.55 | 13.09±2.93 | 12.05±2.96 | 14.08±2.30 | 12.90 ^b^ | <0.001 |
| Illness duration (months) | 27.85±33.02 | 60.80±65.51 | 47.69±73.50 | - | 9.14 ^b^ | <0.001 |
| CPZ | 434.77±218.58 | 226.13±246.48 | 27.31±79.72 | - | 64.38 ^b^ | <0.001 |
| FLU | 0.72±3.84 | 11.28±14.71 | 28.00±19.13 | - | 83.63 ^b^ | <0.001 |
| SANS | 28.29±24.80 | - | - | - | - | - |
| SAPS | 20.83±15.55 | - | - | - | - | - |
| BPRS | 36.62±10.76 | 26.35±7.13 | 29.35±5.55 | - | 25.97 ^b^ | <0.001 |
| YMRS | - | 5.76±8.46 | 2.33±2.52 | - | 6.72 ^c^ | 0.011 |
| HAMD | - | 12.73±9.38 | 21.84±4.52 | - | 35.88 ^c^ | <0.001 |
| WAIS_Information | 16.32±4.80 | 19.53±4.63 | 17.85±5.87 | 20.84±4.59 | 10.11 ^b^ | <0.001 |
| WAIS_Digit symbol | 65.15±15.79 | 67.41±16.85 | 68.48±19.20 | 89.49±12.88 | 34.60 ^b^ | <0.001 |
| Verbal fluency | 14.34±4.30 | 17.62±4.67 | 16.61±4.78 | 19.63±5.13 | 12.27 ^b^ | <0.001 |
| 0-back target accuracy | 0.77±0.28 | 0.88±0.16 | 0.90±0.15 | 0.92±0.13 | 8.37 ^b^ | <0.001 |
| 0-back target reaction time | 556.66±135.86 | 548.93±125.72 | 545.40±110.93 | 484.97±88.03 | 5.59 ^b^ | 0.001 |
| 2-back target accuracy | 0.50±0.25 | 0.60±0.27 | 0.65±0.22 | 0.74±0.19 | 14.48 ^b^ | <0.001 |
| 2-back target reaction time | 707.57±188.93 | 744.20±213.45 | 717.13±176.54 | 650.43±194.61 | 5.00 ^b^ | 0.002 |

Note: Quantitative data were presented as mean ± standard deviation.

Abbreviations: SCZ, Schizophrenia; BD, bipolar disorder; MDD, major depressive disorder patients; HCs, healthy controls; CPZ, chlorpromazine equivalent dose (Leucht et al., 2015); FLU, fluoxetine equivalents dose (Hayasaka et al., 2015); SANS, Scale for the Assessment of Positive Symptoms; SAPS, Scale for the Assessment of Negative Symptoms; BPRS, Brief Psychiatric Rating Scale; YMRS, Young Mania Rating Scale; HAMD, Hamilton Depression Rating Scale.

^a^ χ^2^ test

^b^ One-way ANOVA

^c^ Two-sample t-test

**Table S4** Demographic, clinical, and cognitive characteristics of the group from Dataset #2

| Variables | MDD (n=48) |
| --- | --- |
| Gender (Male/Female) | 19/29 |
| Age (years) | 23.98±5.54 |
| Education (years) | 13.97±2.06 |
| Illness duration (months) | 34.65±35.65 |
| CPZ | 3.47±24.06 |
| FLU | 6.03±15.11 |
| BPRS | 35.15±7.12 |
| HAMD | 21.10±4.18 |
| HAMA | 16.48±6.80 |

Note: Quantitative data were presented as mean ± standard deviation.

Abbreviations: MDD, major depressive disorder patients; CPZ, chlorpromazine equivalent dose (Leucht et al., 2015); FLU, fluoxetine equivalents dose (Hayasaka et al., 2015); BPRS, Brief Psychiatric Rating Scale; HAMD, Hamilton Depression Rating Scale; HAMA, Hamilton Anxiety Rating Scale

**Table S5** Significant differences in static and dynamic RFE among SCZ, BD without psychotic features, MDD without psychotic features, and HCs

| **Regions** | **One-way ANCOVA** | | | | | | |  |  | **Post-hoc analysis** | | | |
| --- | --- | --- | --- | --- | --- | --- | --- | --- | --- | --- | --- | --- | --- |
|  | **k** | **MNI** | | | ***F*_3, 303_** | | ***p*_FDR_** | ***η*^2^** |  | **Comparison** | ***t*** | ***p*_FDR_** | **Cohen’s *d*** |
|  |  | **X** | **Y** | **Z** |  |  |  |  |  |  |  |  |  |
| Static RFE |  |  |  |  |  | |  |  |  |  |  |  |  |
| L ITG | 111 | -48 | -45 | -30 | | 44.38 | <0.001 | 0.31 |  | SCZ>MDDNP | 10.12 | <0.001 | 1.62 |
|  |  |  |  |  |  | |  |  |  | BDNP>MDDNP | 10.40 | <0.001 | 1.98 |
|  |  |  |  |  |  | |  |  |  | MDDNP<HCs | -10.43 | <0.001 | 1.83 |
| L THA | 227 | 0 | -27 | 9 | 41.49 | | <0.001 | 0.29 |  | SCZ>BDNP | 4.14 | <0.001 | 0.66 |
|  |  |  |  |  |  | |  |  |  | SCZ>MDDNP | 10.35 | <0.001 | 1.66 |
|  |  |  |  |  |  | |  |  |  | SCZ>HCs | 4.47 | 0.004 | 0.67 |
|  |  |  |  |  |  | |  |  |  | BDNP>MDDNP | 7.77 | <0.001 | 1.48 |
|  |  |  |  |  |  | |  |  |  | MDDNP<HCs | -10.65 | <0.001 | 1.86 |
| R CAU | 39 | 0 | -27 | 9 | 41.49 | | <0.001 | 0.29 |  | SCZ>BDNP | 5.83 | <0.001 | 0.93 |
|  |  |  |  |  |  | |  |  |  | SCZ>MDDNP | 10.35 | <0.001 | 1.66 |
|  |  |  |  |  |  | |  |  |  | SCZ>HCs | 4.47 | 0.004 | 0.67 |
|  |  |  |  |  |  | |  |  |  | BDNP>MDDNP | 7.77 | <0.001 | 1.48 |
|  |  |  |  |  |  | |  |  |  | MDDNP<HCs | -10.65 | <0.001 | 1.86 |
| R CPL | 253 | 45 | -45 | -33 | | 40.23 | <0.001 | 0.28 |  | SCZ>MDDNP | 10.54 | <0.001 | 1.69 |
|  |  |  |  |  |  | |  |  |  | BDNP>MDDNP | 9.39 | <0.001 | 1.78 |
|  |  |  |  |  |  | |  |  |  | MDDNP<HCs | -9.61 | <0.001 | 1.68 |
| L MOG | 235 | -15 | -96 | 6 | 36.00 | | <0.001 | 0.26 |  | SCZ<MDDNP | -9.85 | <0.001 | 1.58 |
|  |  |  |  |  |  | |  |  |  | BDNP<MDDNP | -9.07 | <0.001 | 1.72 |
|  |  |  |  |  |  | |  |  |  | MDDNP>HCs | 8.99 | <0.001 | 1.57 |
| L LING | 45 | -15 | -96 | 6 | 36.00 | | <0.001 | 0.26 |  | SCZ<MDDNP | -9.85 | <0.001 | 1.58 |
|  |  |  |  |  |  | |  |  |  | MDDNP>HCs | 8.99 | <0.001 | 1.57 |
| R LING | 71 | 21 | -90 | -3 | 23.72 | | <0.001 | 0.19 |  | SCZ<MDDNP | -7.82 | <0.001 | 1.25 |
|  |  |  |  |  |  | |  |  |  | BDNP<MDDNP | -7.77 | <0.001 | 1.48 |
|  |  |  |  |  |  | |  |  |  | MDDNP>HCs | 7.09 | <0.001 | 1.24 |
| L MeFG | 67 | -1 | 54 | -18 | | 23.17 | <0.001 | 0.19 |  | SCZ>MDDNP | 7.55 | <0.001 | 1.21 |
|  |  |  |  |  |  | |  |  |  | BDNP>MDDNP | 7.30 | <0.001 | 1.39 |
|  |  |  |  |  |  | |  |  |  | MDDNP<HCs | -7.56 | <0.001 | 1.32 |
| L IPL | 140 | -12 | -72 | 51 | 21.90 | | <0.001 | 0.18 |  | SCZ<BDNP | -5.57 | <0.001 | 0.89 |
|  |  |  |  |  |  | |  |  |  | SCZ<MDDNP | -7.22 | <0.001 | 1.16 |
|  |  |  |  |  |  | |  |  |  | BDNP<MDDNP | -6.53 | <0.001 | 1.24 |
|  |  |  |  |  |  | |  |  |  | MDDNP>HCs | 8.08 | <0.001 | 1.41 |
| L PoCG | 102 | -12 | -72 | 51 | 21.90 | | <0.001 | 0.18 |  | SCZ<BDNP | -5.57 | <0.001 | 0.89 |
|  |  |  |  |  |  | |  |  |  | SCZ<MDDNP | -7.22 | <0.001 | 1.16 |
|  |  |  |  |  |  | |  |  |  | BDNP<MDDNP | -6.53 | <0.001 | 1.24 |
| R SMA | 35 | 12 | 3 | 63 | 21.17 | | <0.001 | 0.17 |  | SCZ<MDDNP | -6.23 | <0.001 | 1.00 |
|  |  |  |  |  |  | |  |  |  | BDNP<MDDNP | -4.96 | <0.001 | 0.94 |
|  |  |  |  |  |  | |  |  |  | MDDNP>HCs | 7.91 | <0.001 | 1.38 |
| R SPL | 118 | 12 | -69 | 54 | 20.74 | | <0.001 | 0.17 |  | SCZ<MDDNP | -7.23 | <0.001 | 1.16 |
|  |  |  |  |  |  | |  |  |  | BDNP<MDDNP | -5.88 | <0.001 | 1.12 |
|  |  |  |  |  |  | |  |  |  | MDDNP>HCs | 7.58 | <0.001 | 1.33 |
| R MFG | 44 | 42 | -6 | 51 | 15.93 | | <0.001 | 0.14 |  | SCZ<MDDNP | -6.33 | <0.001 | 1.01 |
|  |  |  |  |  |  | |  |  |  | BDNP<MDDNP | -5.18 | <0.001 | 0.98 |
|  |  |  |  |  |  | |  |  |  | MDDNP>HCs | 6.62 | <0.001 | 1.16 |
| L STG | 22 | -57 | 9 | -12 | | 14.54 | <0.001 | 0.13 |  | SCZ>MDDNP | 5.34 | <0.001 | 0.85 |
|  |  |  |  |  |  | |  |  |  | BDNP>MDDNP | 6.03 | <0.001 | 1.15 |
|  |  |  |  |  |  | |  |  |  | MDDNP<HCs | -6.50 | <0.001 | 1.14 |
| L IFG | 20 | -48 | 45 | 3 | 14.39 | | <0.001 | 0.12 |  | SCZ>MDDNP | 6.31 | <0.001 | 1.01 |
|  |  |  |  |  |  | |  |  |  | SCZ>HCs | 4.27 | <0.001 | 0.64 |
|  |  |  |  |  |  | |  |  |  | BDNP>MDDNP | 5.00 | <0.001 | 0.95 |
|  |  |  |  |  |  | |  |  |  | MDDNP<HCs | -3.75 | <0.001 | 0.66 |
| Dynamic RFE | |  |  |  |  | |  |  |  |  |  |  |  |
| R CPL | 586 | 24 | -87 | -51 | | 28.30 | <0.001 | 0.22 |  | SCZ>BDNP | 3.52 | 0.006 | 0.56 |
|  |  |  |  |  |  | |  |  |  | SCZ<MDDNP | -8.46 | <0.001 | 1.35 |
|  |  |  |  |  |  | |  |  |  | BDNP<MDDNP | -8.72 | <0.001 | 1.66 |
|  |  |  |  |  |  | |  |  |  | MDDNP>HCs | 7.54 | <0.001 | 1.32 |
| R PreCG | 32 | 63 | 6 | 36 | 14.88 | | <0.001 | 0.13 |  | SCZ<MDDNP | -5.77 | <0.001 | 0.92 |
|  |  |  |  |  |  | |  |  |  | BDNP<MDDNP | -6.35 | <0.001 | 1.21 |
|  |  |  |  |  |  | |  |  |  | MDDNP>HCs | 5.82 | <0.001 | 1.02 |
| R MFG | 194 | -3 | 30 | -27 | | 14.82 | <0.001 | 0.13 |  | SCZ>BDNP | 3.63 | 0.005 | 0.58 |
|  |  |  |  |  |  | |  |  |  | SCZ>MDDNP | 4.71 | <0.001 | 0.75 |
|  |  |  |  |  |  | |  |  |  | BDNP>MDDNP | 6.49 | <0.001 | 1.23 |
| L MCC | 108 | -24 | -45 | 42 | 11.87 | | <0.001 | 0.11 |  | SCZ>MDDNP | 5.60 | <0.001 | 0.90 |
|  |  |  |  |  |  | |  |  |  | BDNP>MDDNP | 4.96 | <0.001 | 0.94 |
|  |  |  |  |  |  | |  |  |  | MDDNP<HCs | -5.38 | <0.001 | 0.94 |
| L IFGorb | 28 | -48 | 39 | -15 | | 11.29 | <0.001 | 0.10 |  | SCZ>MDDNP | 4.93 | <0.001 | 0.79 |
|  |  |  |  |  |  | |  |  |  | SCZ<HCs | -5.69 | <0.001 | 0.85 |
|  |  |  |  |  |  | |  |  |  | BDNP>MDDNP | 4.03 | 0.001 | 0.77 |
|  |  |  |  |  |  | |  |  |  | MDDNP<HCs | -4.84 | <0.001 | 0.85 |
| L PHG | 20 | -18 | -9 | -6 | 10.90 | | <0.001 | 0.10 |  | SCZ>BDNP | 4.32 | 0.002 | 0.69 |
|  |  |  |  |  |  | |  |  |  | BDNP>MDDNP | 5.19 | <0.001 | 0.99 |
|  |  |  |  |  |  | |  |  |  | BDNP<HCs | -4.78 | 0.004 | 0.86 |
|  |  |  |  |  |  | |  |  |  | MDDNP<HCs | -5.58 | <0.001 | 0.98 |
| L ITG | 43 | -18 | -12 | -42 | | 10.75 | <0.001 | 0.10 |  | SCZ>MDDNP | 4.41 | <0.001 | 0.71 |
|  |  |  |  |  |  | |  |  |  | BDNP>MDDNP | 4.11 | 0.001 | 0.78 |
|  |  |  |  |  |  | |  |  |  | MDDNP<HCs | -5.75 | <0.001 | 1.01 |
| R IFG | 31 | 57 | 21 | -3 | 10.30 | | <0.001 | 0.09 |  | SCZ>BDNP | 4.19 | 0.002 | 0.67 |
|  |  |  |  |  |  | |  |  |  | BDNP>MDDNP | 4.81 | <0.001 | 0.91 |
|  |  |  |  |  |  | |  |  |  | MDDNP<HCs | -5.50 | <0.001 | 0.96 |
| L SFG | 28 | -9 | 0 | 78 | 9.36 | | <0.001 | 0.08 |  | SCZ>BDNP | 3.52 | 0.006 | 0.56 |
|  |  |  |  |  |  | |  |  |  | SCZ<MDDNP | -4.45 | <0.001 | 0.71 |
|  |  |  |  |  |  | |  |  |  | BDNP<MDDNP | -4.44 | <0.001 | 0.84 |
|  |  |  |  |  |  | |  |  |  | MDDNP>HCs | 5.21 | <0.001 | 0.91 |
| L MFG | 115 | -9 | 27 | 45 | 8.50 | | <0.001 | 0.08 |  | SCZ>MDDNP | 4.71 | <0.001 | 0.75 |
|  |  |  |  |  |  | |  |  |  | BDNP>MDDNP | 4.74 | <0.001 | 0.90 |
|  |  |  |  |  |  | |  |  |  | MDDNP<HCs | -4.82 | <0.001 | 0.84 |

Abbreviations: FDR, false discovery rate correction; SCZ, schizophrenia; BDNP, bipolar disorder without psychotic features; MDDNP, major depressive disorder patients without psychotic features; HCs, healthy controls; k, cluster extension in number of voxels; MNI, Montreal Neurological Institute; L, left; R, right; ITG, inferior temporal gyrus; THA, thalamus; CAU, caudate; CPL, cerebellum posterior lobe; MOG, middle occipital gyrus; LING, lingual gyrus; MeFG, medial frontal gyrus; IPL, inferior parietal lobe; PoCG, postcentral gyrus; SMA, supplementary motor area; SPL, superior parietal lobe; MFG, middle frontal gyrus; STG, superior temporal gyrus; IFG, inferior frontal gyrus; PreCG, precentral gyrus; MCC, middle cingulate cortex; IFGorb, inferior frontal gyrus pars orbitalis; PHG, parahippocampal gyrus; SFG, superior frontal gyrus.

**Table S6** Correlation between abnormal static and dynamic RFE and illness duration

| **Regions** | ***r*** | ***p*** |
| --- | --- | --- |
| **Static RFE** |  |  |
| R CAU | -0.06 | 0.345 |
| L PoCG | -0.08 | 0.180 |
| L IFG | -0.15 | 0.015 |
| L IPL | 0.08 | 0.198 |
| L STG | 0.02 | 0.780 |
| L MCC | 0.04 | 0.502 |
| L MFG | -0.04 | 0.507 |
| L LING | 0.04 | 0.526 |
| L PreCG | 0.00 | 0.981 |
| R LING | -0.02 | 0.716 |
| L SFG | -0.05 | 0.382 |
| **Dynamic RFE** |  |  |
| L PIC | 0.01 | 0.828 |
| L PHG | -0.04 | 0.527 |
| R ITG | -0.05 | 0.443 |
| L PoCG | 0.01 | 0.935 |

Abbreviations: L, left; R, right; CAU, caudate; PoCG, postcentral gyrus; IFG, inferior frontal gyrus; IPL, inferior parietal lobe; STG, superior temporal gyrus; MCC, middle cingulate cortex; MFG, middle frontal gyrus; LING, lingual gyrus; PreCG, precentral gyrus; SFG, superior frontal gyrus; PI, posterior insula; PHG, parahippocampal gyrus; ITG, inferior temporal gyrus.


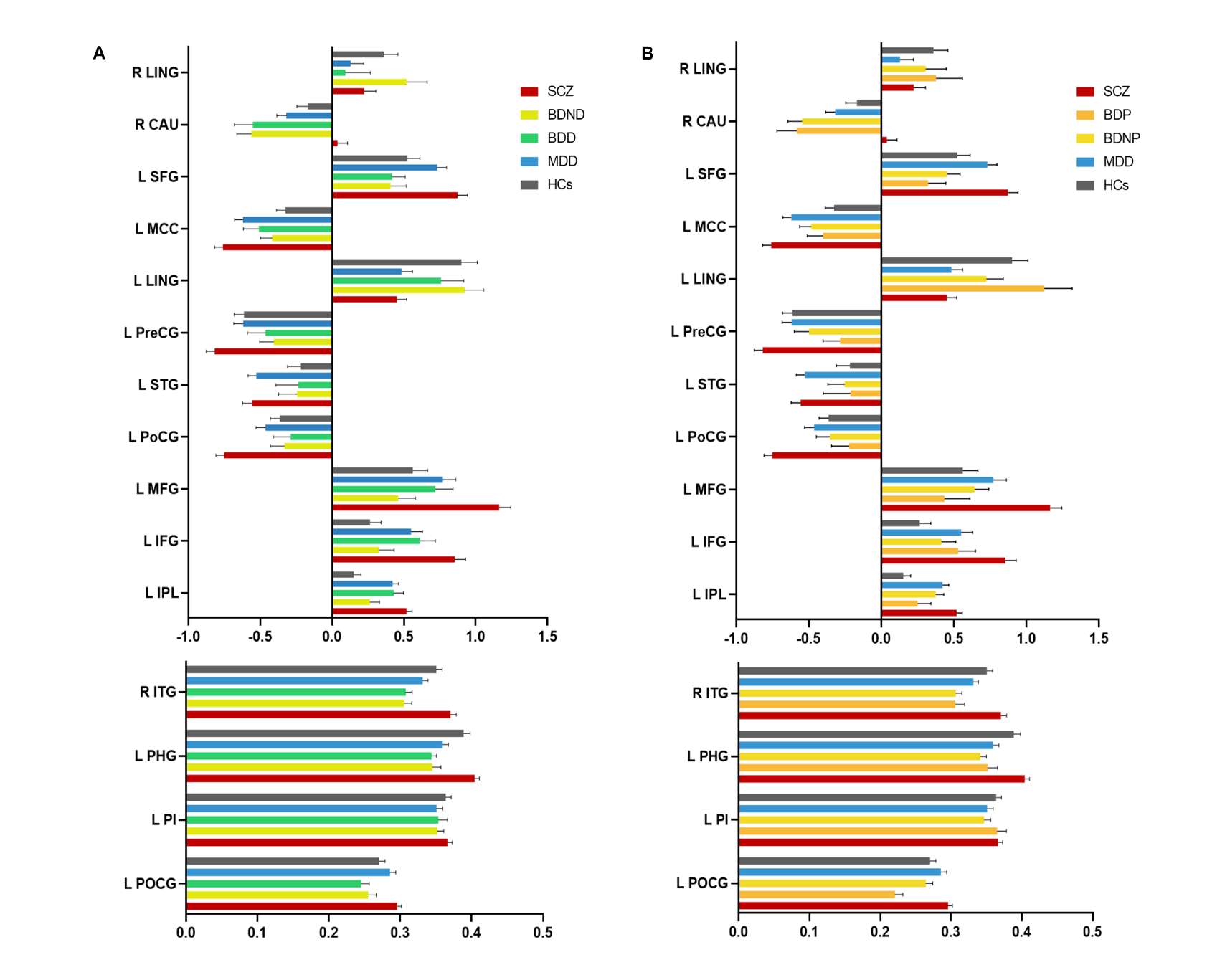
Figure S1 Static and dynamic RFE in significant regions among SCZ, BD subgroups, MDD, and HCs. The bar graphs (A and B) depict the static RFE (upper graphs) and dynamic RFE (lower graphs) among SCZ, BD subgroups, MDD, and HCs. SCZ, schizophrenia; BDND, bipolar disorder in a non-depressive state; BDD, bipolar disorder in a depressive state; BDP, bipolar disorder with psychotic features; BDNP, bipolar disorder without psychotic features; MDD, major depressive disorder patients; HCs, healthy controls; L, left; R, right; IPL, inferior parietal lobule; IFG, inferior frontal gyrus; MFG, middle frontal gyrus; PoCG, postcentral gyrus; STG, superior temporal gyrus; PreCG, precentral gyrus; LING, lingual gyrus; MCC, middle cingulate cortex; SFG, superior frontal gyrus; CAU, caudate; PI, posterior insula; PHG, parahippocampal gyrus; ITG, inferior temporal gyrus.

**References**

Deng, S., Franklin, C. G., O'Boyle, M., Zhang, W., Heyl, B. L., Jerabek, P. A., . . . Fox, P. T. (2022). Hemodynamic and metabolic correspondence of resting-state voxel-based physiological metrics in healthy adults. *Neuroimage, 250*, 118923. doi:10.1016/j.neuroimage.2022.118923

Hayasaka, Y., Purgato, M., Magni, L. R., Ogawa, Y., Takeshima, N., Cipriani, A., . . . Furukawa, T. A. (2015). Dose equivalents of antidepressants: Evidence-based recommendations from randomized controlled trials. *J Affect Disord, 180*, 179-184. doi:10.1016/j.jad.2015.03.021

Keller, J., Gomez, R., Williams, G., Lembke, A., Lazzeroni, L., Murphy, G. M., Jr., & Schatzberg, A. F. (2017). HPA axis in major depression: cortisol, clinical symptomatology and genetic variation predict cognition. *Mol Psychiatry, 22*(4), 527-536. doi:10.1038/mp.2016.120

Leucht, S., Samara, M., Heres, S., Patel, M. X., Furukawa, T., Cipriani, A., . . . Davis, J. M. (2015). Dose Equivalents for Second-Generation Antipsychotic Drugs: The Classical Mean Dose Method. *Schizophr Bull, 41*(6), 1397-1402. doi:10.1093/schbul/sbv037

Li, Z., Kadivar, A., Pluta, J., Dunlop, J., & Wang, Z. (2012). Test-retest stability analysis of resting brain activity revealed by blood oxygen level-dependent functional MRI. *J Magn Reson Imaging, 36*(2), 344-354. doi:10.1002/jmri.23670

Li, Z., Zhu, Y., Childress, A. R., Detre, J. A., & Wang, Z. (2012). Relations between BOLD fMRI-derived resting brain activity and cerebral blood flow. *PLoS One, 7*(9), e44556. doi:10.1371/journal.pone.0044556

Lurie, D. J., Kessler, D., Bassett, D. S., Betzel, R. F., Breakspear, M., Kheilholz, S., . . . Calhoun, V. D. (2020). Questions and controversies in the study of time-varying functional connectivity in resting fMRI. *Netw Neurosci, 4*(1), 30-69. doi:10.1162/netn_a_00116

Lv, H., Wang, Z., Tong, E., Williams, L. M., Zaharchuk, G., Zeineh, M., . . . Wintermark, M. (2018). Resting-State Functional MRI: Everything That Nonexperts Have Always Wanted to Know. *AJNR Am J Neuroradiol, 39*(8), 1390-1399. doi:10.3174/ajnr.A5527

Magnuson, M. E., Thompson, G. J., Pan, W. J., & Keilholz, S. D. (2014). Effects of severing the corpus callosum on electrical and BOLD functional connectivity and spontaneous dynamic activity in the rat brain. *Brain Connect, 4*(1), 15-29. doi:10.1089/brain.2013.0167

Majeed, W., Magnuson, M., Hasenkamp, W., Schwarb, H., Schumacher, E. H., Barsalou, L., & Keilholz, S. D. (2011). Spatiotemporal dynamics of low frequency BOLD fluctuations in rats and humans. *Neuroimage, 54*(2), 1140-1150. doi:10.1016/j.neuroimage.2010.08.030

Matsui, T., Murakami, T., & Ohki, K. (2019). Neuronal Origin of the Temporal Dynamics of Spontaneous BOLD Activity Correlation. *Cerebral Cortex, 29*(4), 1496-1508. doi:10.1093/cercor/bhy045

Power, J. D., Barnes, K. A., Snyder, A. Z., Schlaggar, B. L., & Petersen, S. E. (2012). Spurious but systematic correlations in functional connectivity MRI networks arise from subject motion. *Neuroimage, 59*(3), 2142-2154. doi:10.1016/j.neuroimage.2011.10.018

Sato, J. R., Biazoli, C. E., Jr., Moura, L. M., Crossley, N., Zugman, A., Picon, F. A., . . . Jackowski, A. P. (2019). Association Between Fractional Amplitude of Low-Frequency Spontaneous Fluctuation and Degree Centrality in Children and Adolescents. *Brain Connect, 9*(5), 379-387. doi:10.1089/brain.2018.0628

Thompson, G. J. (2018). Neural and metabolic basis of dynamic resting state fMRI. *Neuroimage, 180*, 448-462. doi:10.1016/j.neuroimage.2017.09.010

Yan, C. G., Wang, X. D., Zuo, X. N., & Zang, Y. F. (2016). DPABI: Data Processing & Analysis for (Resting-State) Brain Imaging. *Neuroinformatics, 14*(3), 339-351. doi:10.1007/s12021-016-9299-4

Yang, G. J., Murray, J. D., Repovs, G., Cole, M. W., Savic, A., Glasser, M. F., . . . Anticevic, A. (2014). Altered global brain signal in schizophrenia. *Proc Natl Acad Sci U S A, 111*(20), 7438-7443. doi:10.1073/pnas.1405289111

Yang, J., Tao, H., Sun, F., Fan, Z., Yang, J., Liu, Z., . . . Chen, X. (2023). The anatomical networks based on probabilistic structurally connectivity in bipolar disorder across mania, depression, and euthymic states. *J Affect Disord, 329*, 42-49. doi:10.1016/j.jad.2023.02.109
